# Impact of Soilless Growing Media on Growth, Yield, Fruit Quality, and Root‐Knot Nematode Incidence of Cucumber Under Protected Cultivation

**DOI:** 10.1002/pei3.70196

**Published:** 2026-08-03

**Authors:** S. K. Acharya, G. S. Patel, Lalu Prasad Yadav, Anand Sahil, Prashant Kaushik, N. K. Meena, Prakash Mahala, Ali Khadivi

**Affiliations:** ^1^ College of Horticulture Sardarkrushinagar Dantiwada Agricultural University Mehsana Gujarat India; ^2^ ICAR‐Central Institute for Arid Horticulture (CIAH) Bikaner Rajasthan India; ^3^ ICAR‐Central Horticultural Experiment Station (CIAH RS) Godhra Gujarat India; ^4^ Chaudhary Charan Singh Haryana Agricultural University Hisar Haryana India; ^5^ College of Horticulture and Forestry Agriculture University, Kota (AU) Jhalawar Rajasthan India; ^6^ Regional Research Station Punjab Agricultural University Abohar India; ^7^ Department of Horticultural Sciences, Faculty of Agriculture and Natural Resources Arak University Arak Iran

**Keywords:** cocopeat, *Cucumis sativus*, soilless culture, sustainable horticulture, vermiculite

## Abstract

Soilless cultivation has emerged as an effective approach for improving cucumber productivity under protected cultivation by enhancing root‐zone conditions and minimizing soil‐borne constraints. However, the performance of different growing media combinations remains insufficiently understood. Therefore, this study investigated the effects of different growing media on cucumber growth, yield, fruit quality, and root‐knot nematode incidence under protected cultivation. A completely randomized design (CRD) experiment was conducted with 13 different growing media treatments, including soil, perlite, vermiculite, cocopeat, sand, and their combinations. According to the findings, the Vermiculite + Cocopeat in the ratio of 1:1 (T9) combination reduced root‐knot nematode infection while dramatically increasing vine length, fruit set, fruit retention, fruit yield (28.05 kg/m^2^), and quality metrics. Vine length and yield were found to be strongly positively correlated by correlation analysis, while productivity was negatively correlated with root‐knot infestation. Fruit set, flowering period, and fruit retention all directly increased yield, according to path analysis. Strong genetic control over these variables was shown by estimates of variance components, which showed moderate heritability for fruit set and retention and high heritability (114.18%) for days to flower initiation. However, yield‐related traits exhibited low heritability *viz* yield per vine (14.24%) and YPP (12.89%), highlighting the significant role of environmental factors. These findings suggest that combination of Vermiculite and Cocopeat is the most suitable growing medium for cucumber cultivation in protected conditions, optimizing yield and reducing disease susceptibility.

## 1 Introduction

Cucumber ( *Cucumis sativus* L.), a member of the Cucurbitaceae family, is a widely cultivated vegetable crop known for its high economic value and nutritional benefits (Yadav et al. 2019). It is an essential component of protected cultivation systems, where optimal environmental conditions such as temperature, humidity, and light availability significantly enhance its growth and productivity (Singh et al. 2017). The adoption of greenhouse and soilless cultivation techniques has gained popularity due to their potential to increase yield, improve fruit quality, and minimize soil‐borne diseases (Albaho et al. 2008; Gashgari et al. 2018; Hussain et al. 2014; Kumar et al. 2022; Mazahreh et al. 2015; Raviv et al. 2008; Sahil, Singh, Yadav, et al. 2026).

Because it affects water retention, nutritional availability, and root development, growing media are essential to the production of cucumbers. Compaction of the soil, inadequate aeration, and vulnerability to root‐knot nematodes and other infections are some of the difficulties associated with traditional soil‐based farming (Dhillon et al. 2022). Root‐knot nematodes (Meloidogyne spp.) are among the most destructive soil‐borne pathogens affecting cucumber production worldwide, particularly under protected cultivation where continuous cropping often leads to a rapid buildup of nematode populations. Infection by Meloidogyne spp. causes root galling, impairs water and nutrient uptake, reduces plant vigor, and results in substantial yield and quality losses. The physical and biological properties of growing media can influence nematode survival, movement, and root infection by altering aeration, moisture availability, and root‐zone conditions. Consequently, selecting suitable soilless substrates may not only improve plant growth and productivity but also reduce root‐knot nematode infestation, contributing to more sustainable cucumber production under protected cultivation. Alternative substrates like sand, perlite, vermiculite, and cocopeat are being used more and more in protected settings to get around these restrictions (Sarkar and Majumder 2019). It has been demonstrated that these soilless media improve nutrient uptake, maximize water use efficiency, and offer a growing environment devoid of pathogens (He et al. 2021).

Previous studies have demonstrated that the physical and hydraulic properties of soilless substrates strongly influence cucumber growth, nutrient uptake, water availability, and root‐zone aeration under protected cultivation. Organic substrates such as cocopeat and inorganic materials including vermiculite and perlite have been widely investigated because of their favorable water‐holding capacity, aeration, and structural stability (Alifar et al. 2010; Ghehsareh and Kalbasi 2012; Ghehsareh et al. 2012; Lizhong et al. 2022; Sahil et al. 2023). However, the comparative performance of different substrate combinations, particularly with respect to plant growth, fruit yield, fruit quality, and root‐knot nematode incidence under naturally ventilated protected cultivation, remains insufficiently understood. One of the most damaging soil‐borne diseases limiting cucumber production is root‐knot nematodes, especially in protected cultivation where frequent cropping and ideal environmental conditions encourage their quick proliferation. Reduced plant vigor, fruit yield, and quality are the results of these nematodes' induction of root galls, which hinder water and nutrient uptake. Nematode establishment and disease severity may be impacted by the physical characteristics of soilless substrates, such as aeration, porosity, moisture retention, and drainage, which directly affect root‐zone conditions and root health. Therefore, in order to ascertain whether various growing media could contribute to better root health and more sustainable cucumber production under protected cultivation, the current study evaluated root‐knot nematode incidence in addition to plant growth, yield, and fruit quality (Wan Shafiin et al. 2021).

Even with soilless cultivations benefits, choosing the best growing medium is still very difficult. Plant growth, yield, and resilience to biotic and abiotic stressors can all be impacted by different substrate combinations. Thus, the purpose of this study is to assess how different growing media affect the quality, growth, and yield of cucumbers grown in a protected, naturally ventilated environment. The study aims to provide light on effective and sustainable cucumber production methods by determining the ideal substrate mix.

## 2 Materials and Methods

The experiment was conducted during kharif season of 2020 and 2021 on PPC 2 variety of cucumber in naturally ventilated protected structure erected at College Farm, College of Horticulture, SDAU, Jagudan. Experiment consists 13 treatments of different growing media and their combinations on a volume basis (lxwxh) in 2 × 1 × 1 foot bag size viz., T_1_—Soil, T_2_—Perlite, T_3_—Vermiculite, T_4_—Cocopeat, T_5_ –Sand, T_6_—Perlite and Vermiculite (1:1), T_7_‐ Perlite and Cocopeat (1:1), T_8_—Perlite and Sand (1:1), T_9_—Vermiculite + Cocopeat (1:1), T_10_—Vermiculite and Sand (1:1), T_11_—Cocopeat and Sand (1:1), T_12_—Perlite, Vermiculite and Cocopeat (1:1:1) and T_13_—Perlite, Vermiculite, and Cocopeat and Sand (1:1:1:1). It was laid out in CRD with three repetitions. Fresh growing media were used for all treatments during the first year of the experiment. In the second year, the same growing media were reused to evaluate their performance over successive cropping cycles. The Physico‐chemical properties of substrate are presented in Table 1. As soil and sand were included among the evaluated substrates and root‐knot nematodes are common soil‐borne pathogens affecting cucumber, root‐knot nematode incidence (RKI) was recorded as a common observation across all treatments to compare the influence of different growing media on root health. Recommended dose of fertilizer (RDF) (100/60/60 kg/ha NPK) were applied at the time of filling of growing media on volume basis. A standard nutrient solution containing NPK (19:19:19) along with micronutrients Grade IV@1% (which contains essential nutrients for plants like zinc, iron, boron, copper, manganese and molybdenum) was applied fortnightly. If plants showed deficiency symptoms of the nutrients apart from treatment common application were kept. Ten grow bags (2 × 1 × 1) were used per treatment and they are repeated thrice which contains 30 grow bags per treatment. Seeds of cucumber cv. PPC‐2 were directly sown in the respective grow bags following the standard package of practices. Two seeds were sown per grow bag, and after germination, seedlings were thinned to retain one healthy and uniform plant per grow bag.

**TABLE 1:** Physico‐chemical properties of substrate used in the study.

Sr.No.	Name of substrate	Properties
1.	Soil	loamy sand, comprising 77.85% sand, 13.65% silt, and 7.93% clay, with a pH of 7.79 and an electrical conductivity (EC) of 0.192 dS m^−1^.
2.	Sand	Sand characterized by its good aeration and poor water‐holding capacity, had a pH of 6.5 and water holding capacity is (WHC) about 49%.
3.	Perlite	Perlite, a crystal‐white lightweight substrate produced by heating volcanic material to approximately 1400°C, was included for its excellent aeration properties. It has pH is 8.50, EC‐0.08 dS m^−1^.
4.	Cocopeat	Cocopeat, a lightweight by‐product of the coir industry, exhibited very good water retention and a pH of about 6.7. It also contained small amounts of potassium and showed relatively higher EC values depending on water quality. WHC is about 7 times of its weight.
5.	Vermiculite	Vermiculite, a 2:1 type clay mineral, provided high water‐holding capacity while efficiently draining excess moisture and high in pH is low.

*Source:* Natural Resource Management Laboratory, COH, Jagudan.

Plants of cucumber were irrigated with 2 mm micro tube emitters for half an hour daily to meet out the crop water requirements. There were 60 irrigation were applied during crop grown period and 625 mL water per irrigation was applied (37.5 L water per plant during entire growing season). Plants were trained to a single stem using vertical polypropylene twine attached to overhead support wires. Regular tying and removal of lateral shoots were carried out according to standard protected cucumber cultivation practices. Although the first harvest commenced approximately 35–45 days after sowing, plants were maintained under protected cultivation for nearly 90 days to facilitate continuous harvesting and to monitor root‐knot nematode development throughout the cropping period. Observations were recorded on vine length at 30 days of planting (cm), vine length at 60 days of planting (cm), number of flower per vine, number of fruit set per vine, fruit setting, minimum days to flower initiation and days taken from fruit set to edible maturity, maximum number of fruits per vine, fruit retention, fruit length (cm), fruit Diameter (cm), average fruit weight (g), yield per vine (kg) and yield per m^2^ area (kg), TSS (°brix) and root knot index (RKI). RKI were observed in roots of 03 randomly selected plants at 30, 60 and 90 days after planting and number of juveniles nematodes per 100 cc of soil prior to sowing and post‐harvest of cucumber. Natural nematode infestation were scaled after carefully washed roots and evaluated using the 0–5 galling scale of Taylor and Sasser (1978) and Siddiqui et al. (2014) where 0 = no galls, 1 = 1–10 galls, 2 = 11–30 galls, 3 = 31–70 galls, 4 = 71–100 galls, and 5 = more than 100 galls with severe root deformation. The mean gall number per plant was recorded, and the root‐knot index (RKI) was calculated accordingly. Data were subjected to statistical analysis as per the standard procedure.

### 2.1 Statistical Analysis

Statistical analyses were conducted to assess variability and relationships among traits across different treatments and years. The statistical methods employed enabled a thorough assessment of the data, leading to strong conclusions regarding the genetic and environmental influences on trait performance. The ANOVA results elucidated the significance of factors, including replication, treatments, and residuals on the observed traits. Mean squares, F‐ratios, and significance levels (*** for *p* < 0.001, ** for *p* < 0.01, * for *p* < 0.05) were computed for both quantitative and qualitative traits. Standard errors (S.Em+) and critical differences (C.D. at 5% and 1%) were utilized to effectively compare treatment means (Gomez and Gomez 1984). Estimates of genotypic and phenotypic variance components were obtained for each trait. Broad‐sense heritability (H^2^) is defined as the ratio of genotypic variance to phenotypic variance, expressed as a percentage (Johnson and Wichern 2007). The genetic advance was calculated to evaluate the potential enhancement of traits under selection, offering important insights into the genetic potential and environmental stability of the assessed traits (Johnson et al. 1955). PCA was conducted to decrease dimensionality and pinpoint essential characteristics that contribute to overall variability. Eigenvalues, percentage of variation explained, and cumulative percentage of variance were calculated (Rao 1964). A scree plot and PCA biplot were created to illustrate the clustering of treatments and the interrelationships among traits. The PCA results identified traits that exert the greatest influence on variability (Yan and Kang 2003). Correlation coefficients between traits were computed for the years 2020 and 2021 independently. Heatmaps and half‐triangle correlation matrices were utilized to illustrate the relationships, offering insights into traits exhibiting strong positive or negative correlations over the two‐year period (Wright 1921). The path analysis examined both direct and indirect effects of traits on yield per vine (YPV). A regression‐based method was employed to estimate the direct contributions of individual traits, with results visualized to emphasize the main factors influencing yield performance. Mean values, ranges, coefficients of variation (CV), F‐ratios, and probabilities for key cucumber traits were analyzed and compared between 2020 and 2021. Shared (a, a) or distinct (a, b) letters indicated statistical differences across years for each trait.

## 3 Results

### 3.1 Effect of Growing Media on Growth, Yield and Quality Parameters

Traits such as vine length at 30 days after sowing (VL30), vine length at 60 days after sowing (VL60), total soluble solids (TSS), and yield were significantly influenced by the growing media treatments (Table 2). The ANOVA revealed highly significant treatment effects (*p* < 0.001) for several growth and yield traits, including VL60 and yield (kg m^−2^), indicating that the choice of growing medium substantially affected cucumber performance. Fruit length (FL) was also significantly affected (*p* < 0.001), whereas fruit diameter (FD) showed a significant treatment effect at *p* < 0.01.

**TABLE 2:** Analysis of variance (ANOVA) showing the effects of growing media, year, and their interaction on growth, yield, fruit quality, and root‐knot nematode‐related traits of cucumber grown under protected cultivation during 2020 and 2021.

Sources of variations	Treatments	Year	Year × Treatments	Residual
DFI	1.78	76.19**	1.98	9.02
DFM	0.74	2.01*	0.26	0.44
FD	0.47**	3.42***	0.03	0.15
FL	10.01***	0.03	1.34	1.68
FR	207.6**	41.85	3.26	64.25
FS	351.0***	36.88	20.77	50.85
FW	3073.99***	111.7	106.63	187.95
NFLV	30.87**	27.61	1.84	11.53
NFRV	109.45***	3.38	1.03	4.14
NFSV	98.28***	193.75***	1.81	2.18
RKI	4.1***	0	0.22***	0.01
TSS	0.03*	0.65***	0.01	0.01
VL30	3264.57***	986.92***	27.35	36.19
VL60	4352.78***	2713.96***	199.13	217.32
YPP	354.56***	3.08	6.86	8.35
YPV	3.18***	0.22	0.03	0.09
Y/M^2^	28.27***	0.82	0.64	0.63
Yield/kg/m^2^	28.05***	6.57**	0.75	0.78

*Note:* [Days to flower initiation (DFI), days to first male flowering (DFM), fruit diameter (FD), fruit length (FL), fruit retention (FR), fruit setting (FS), and fruit weight (FW). Reproductive traits were assessed through the number of flowers per vine (NFLV), number of fruits per vine (NFRV), and number of fruit set per vine (NFSV), (RKI), total soluble solids (TSS), vine length at 30 days after planting (VL30) and vine length at 60 days after planting (VL60), Yield parameters included yield per plant (YPP), yield per vine (YPV), and yield per square meter (Y/M^2^)].

Abbreviation: NS = not significant.

* *p* < 0.05.

** *p* < 0.01.

*** *p*ot < 0.001.

The year also had a substantial impact on a number of traits, such as days to first flowering initiation (DFI), VL60, and yield (kg m^−2^), indicating that phenotypic variations were driven by environmental change between the two growing seasons. Additionally, root‐knot infestation index (RKI) and TSS showed significant year × treatment interactions, suggesting that these features responded differently to growth media in different years. For the majority of other attributes, however, the interaction effect was not significant, indicating that treatment performance was largely stable throughout the seasons. The unexplained experimental variance caused by random error and other uncontrollable environmental factors is represented by the residual mean squares.

The ANOVA results show that under protected culture, growing media treatments and seasonal environmental variables had a substantial impact on cucumber growth, yield, fruit quality, and root health. While the strong year effects emphasize the impact of seasonal environmental variability, the significant treatment effects show that crop performance is significantly influenced by substrate composition. The importance of assessing substrate performance throughout several growing seasons is highlighted by the strong treatment × year interactions found for a small number of characteristics, which imply that some responses were impacted by annual environmental conditions. Significant differences in vine length were observed among the growing media treatments. Vermiculite + cocopeat (1:1) (T9) consistently produced the longest vines at both 30 and 60 days after sowing, demonstrating the beneficial effects of balanced water‐holding capacity and aeration on vegetative growth.

### 3.2 Correlation Among the Traits During the Year 2020

**FIGURE 1:**
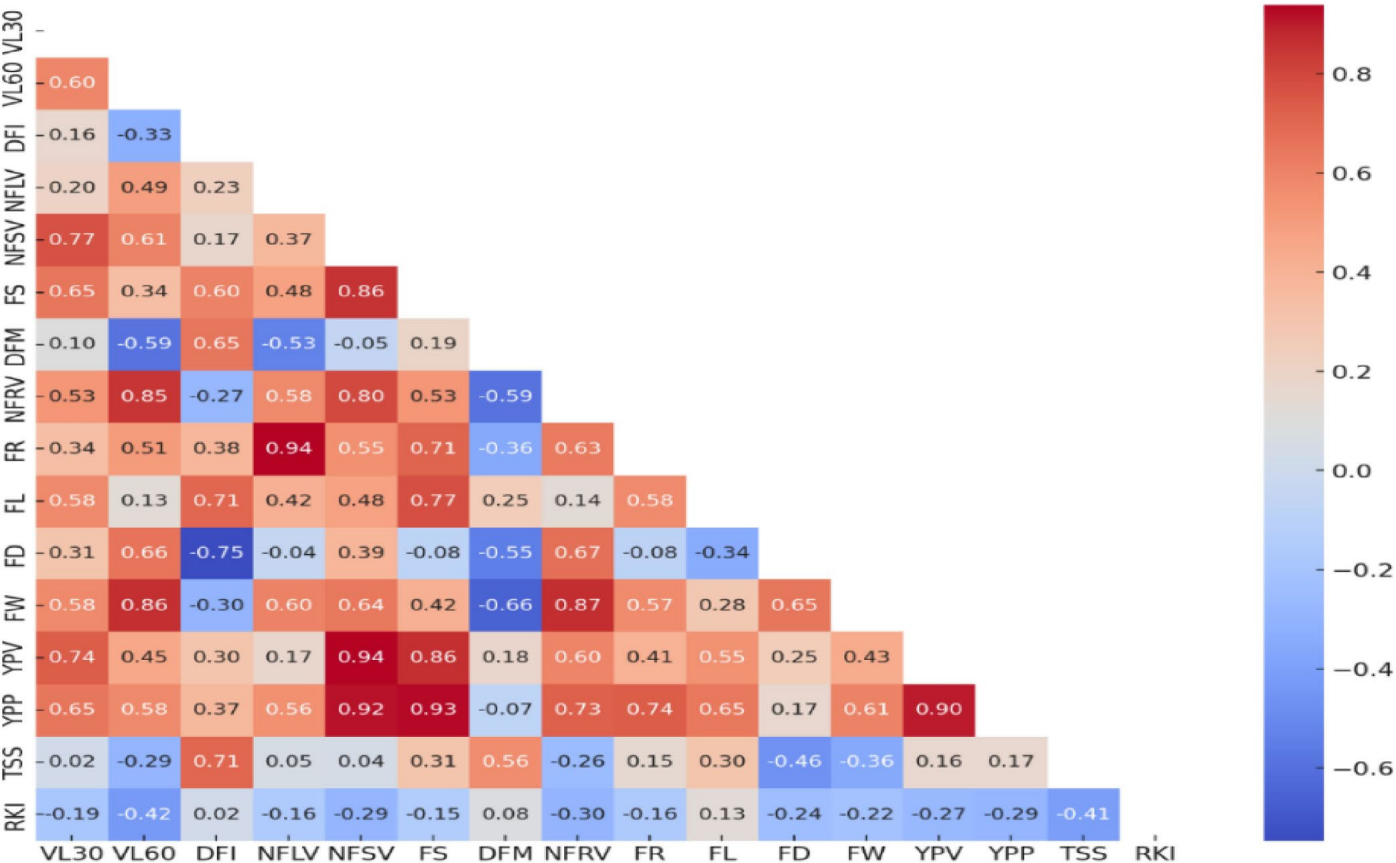
Heatmaps and half‐triangle correlation matrices among different traits of *C. sativus* during the year 2020.

Correlation analysis (Figure 1) revealed clear and biologically meaningful associations among growth, yield, and quality traits during 2020. Early vine vigor (VL30) and vine length at 60 DAS (VL60) showed strong positive correlations with yield attributes, particularly fruit weight, number of fruits, fruit size traits, yield per plant, and yield per unit area, indicating that vegetative growth played a key role in determining productivity. Yield per plant (YPP) and yield per unit area (YPV) were strongly and positively associated with fruit number per vine, fruit size parameters, and fruit weight, confirming their direct contribution to yield improvement.

Fruit set and fruit number traits exhibited strong interrelationships and were positively correlated with yield components, while root‐knot nematode index (RKI) showed a consistent negative association with most growth and yield traits, highlighting the adverse effect of nematode infestation on plant performance. Total soluble solids (TSS) exhibited weak or negative correlations with yield traits, indicating a trade‐off between yield and fruit quality under protected cultivation. Overall, the correlation pattern suggests that improved vine growth, fruit number, and fruit size collectively enhanced cucumber yield, whereas higher nematode incidence negatively influenced productivity.

### 3.3 Correlation Analysis Among the Traits in 2021

**FIGURE 2:**
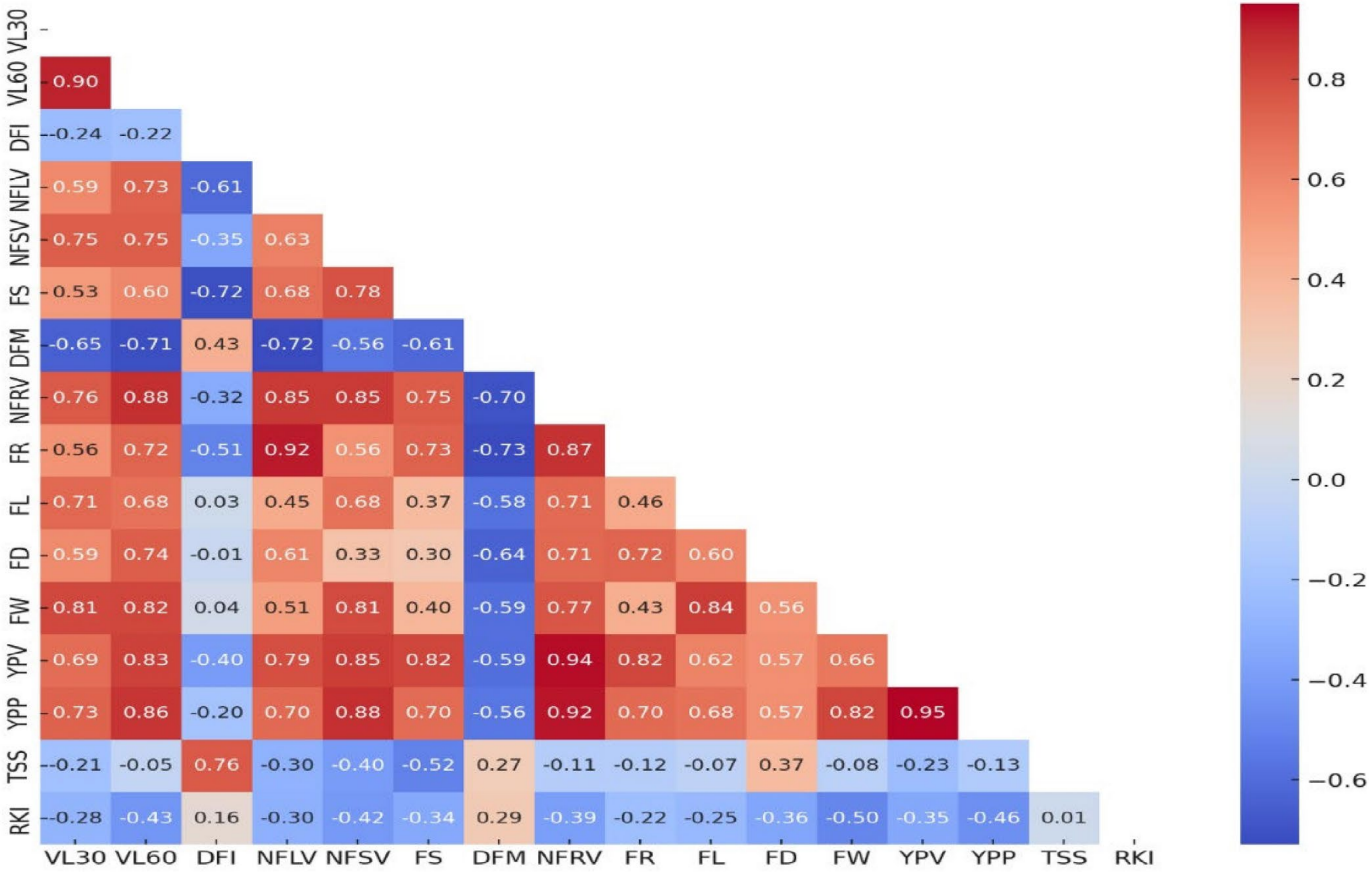
Heatmaps and half‐triangle correlation matrices among different traits of *C. sativus* during the year 2021.

Correlation analysis based on heatmaps and half‐triangle matrices (Figure 2) revealed consistent and biologically meaningful associations among vegetative growth, yield, fruit traits, and root‐knot nematode response during 2021. Vine length at 30 and 60 DAS showed strong positive relationships with major yield components, particularly fruit weight, number of fruits per vine, fruit size traits, yield per plant, and yield per unit area, indicating that vigorous vegetative growth directly contributed to higher productivity. Fruit weight and fruit number traits exhibited strong interrelationships and were the principal contributors to yield expression.

Yield per plant and yield per unit area showed a very strong positive association with fruit number, fruit weight, and fruit size parameters, confirming their central role in determining cucumber yield under protected cultivation. Conversely, the root‐knot nematode index (RKI) exhibited a consistent negative correlation with vegetative growth and yield‐related traits, highlighting the suppressive effect of nematode infestation on crop performance.

Fruit quality traits, particularly total soluble solids (TSS), showed weak or negative correlations with yield traits, suggesting a trade‐off between yield and quality attributes. Days to flowering initiation exhibited a positive association with TSS but negative associations with yield components, indicating that delayed flowering may favor quality traits rather than yield accumulation. Overall, the correlation patterns observed in 2021 closely mirrored those of 2020, with minor variations in correlation strength likely due to seasonal microclimatic differences. These results collectively emphasize the importance of vegetative vigor and fruit number in enhancing yield, while underscoring the negative impact of nematode incidence and the yield–quality trade‐off under protected cultivation.

### 3.4 Principal Component Analysis (PCA)

**FIGURE 3:**
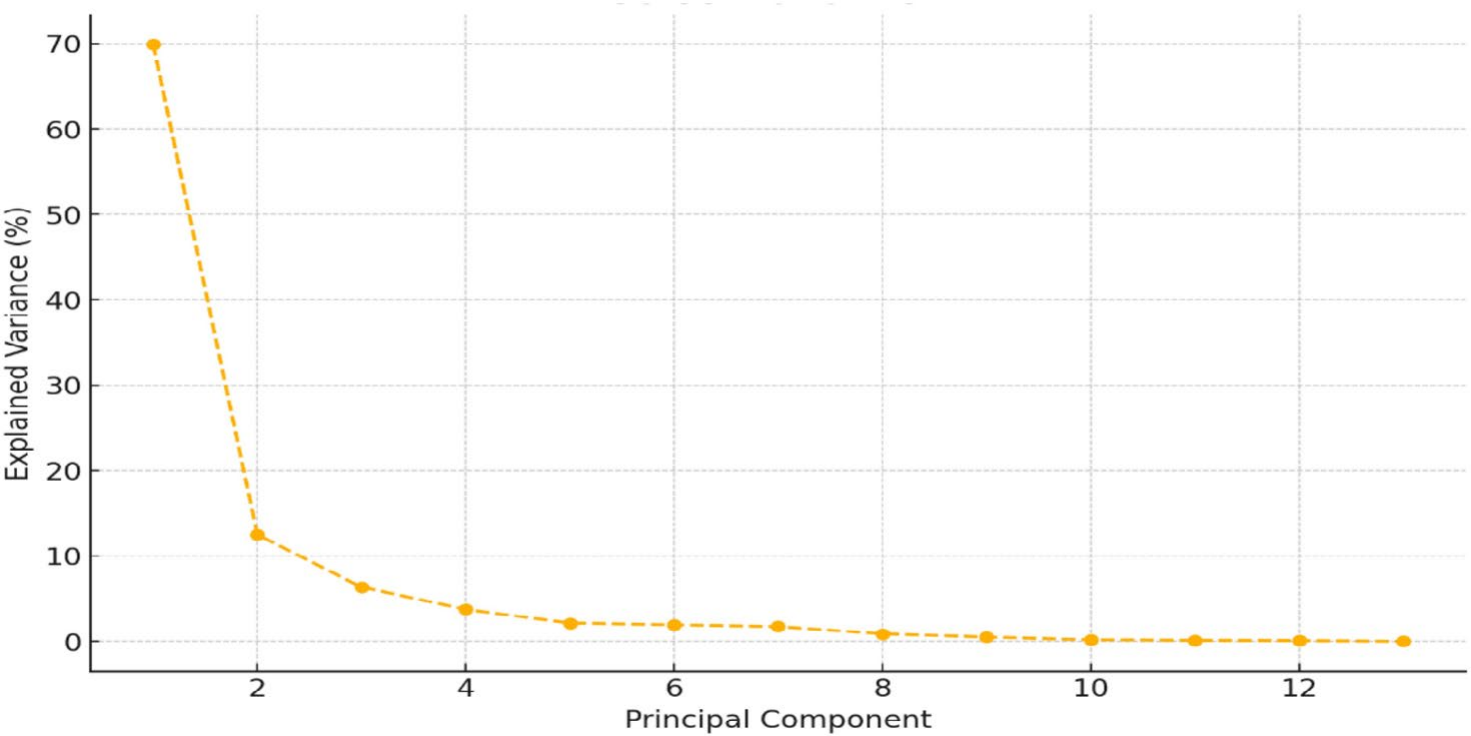
Principal component analysis (PCA) of cucumber traits under different growing media.

Table 3 and Figure 3 showed the results of a Principal Component Analysis (PCA), showing the eigen values, variation explained, and cumulative variance for each principal component (PC). PC1 explains 69.89% of the variance, indicating that it captures the largest portion of the data's variability. The next two components, PC2 and PC3, explain 12.49% and 6.34% respectively, meaning that the first three components together account for 88.72% of the total variance. This highlights that most of the information in the dataset is captured by just the first few principal components.

**TABLE 3:** Principal component analysis (PCA) showing the contribution of growth, yield, fruit quality, and root‐knot nematode‐related traits to the principal components of cucumber grown under different soilless growing media.

Principal Component	Eigenvalue	Variation Explained (%)	Cumulative variance (%)
PC1	12.11	69.89	69.89
PC2	2.16	12.49	82.38
PC3	1.1	6.34	88.72
PC4	0.65	3.73	92.45
PC5	0.37	2.13	94.59
PC6	0.33	1.93	96.52
PC7	0.3	1.72	98.24
PC8	0.15	0.88	99.12
PC9	0.09	0.5	99.61
PC10	0.03	0.18	99.8
PC11	0.02	0.11	99.91
PC12	0.02	0.09	100
PC13	0	0	100

### 3.5 Estimates of Variance Components

The Estimates of Variance Components are central to understanding how genetic and environmental factors contribute to the variation in each trait showed in Table 4. Traits like DFI (114.18%), TSS (95.78%), and DFM (89.84%) show very high heritability, indicating that genetic factors play a dominant role in determining these traits. This implies that selective breeding could effectively improve these traits. Traits like FS (48.6%) and FR (68.55%) have moderate heritability, meaning that both genetic and environmental factors contribute significantly. Traits like YPV (14.24%) and YPP (12.89%) have low heritability, suggesting that environmental factors have a more substantial influence on these traits.

**TABLE 4:** Estimates of variance components of different traits of *Cucumis sativus* .

Traits	Genotypic Variance	Phenotypic Variance	Heritability (%)	Genetic Advance
VL 30	49.18	550.28	8.94	62.68
VL60	252.37	891.4	28.31	449.77
DFI	8.75	7.67	114.18	337.84
NFLV	9.99	13.25	75.44	238.47
NFSV	5.06	19.59	25.82	58.07
FS	45.08	92.76	48.6	326.34
DFM	0.43	0.48	89.84	58.84
NFRV	3.56	20.06	17.74	33.46
FR	52.64	76.79	68.55	497.38
FL	1.59	2.9	54.75	69.01
FD	0.18	0.23	79.98	34.13
FW	171.76	624.06	27.52	360.72
YPV	0.08	0.56	14.24	4.03
YPP	7.99	62	12.89	36.46
TSS	0.02	0.02	95.78	14.49
RKI	0.05	0.68	7.37	1.65

Traits with high genetic advance, such as VL60 (449.77) and FR (497.38), indicate substantial potential for improvement through selective breeding. These traits are likely to respond well to genetic selection. Traits like RKI (1.65) and YPV (4.03) show much lower genetic advance, indicating limited progress through selection. VL60 has a substantial genotypic variance (252.37) compared to its phenotypic variance (891.4), implying that it has a high genetic potential for improvement. In contrast, NFRV (3.56) genotypic variance vs. (20.06) phenotypic variance suggests that environmental factors might have a more significant impact on this trait.

High heritability (traits like VL60, DFI) typically results in higher genetic advance, suggesting that breeding will be highly effective for improving these traits. Traits with moderate heritability (such as FS, NFLV) will respond well to breeding but also require attention to environmental factors. Low heritability traits like YPV and YPP will see minimal genetic improvement unless accompanied by changes in the environment or management practices. Important information about the genetic and environmental factors influencing trait variability was revealed by the estimations of variance components. Strong genetic control over variables like days to flowering initiation (DFI), total soluble solids (TSS), and days to first male flowering (DFM) was demonstrated by their high heritability (> 85%). Fruit set (FS) and fruit retention (FR) showed moderate heritability, indicating a balanced impact of environmental and genetic factors. On the other hand, traits such as yield per plant (YPP) and yield per vine (YPV) showed low heritability (< 20%), suggesting that environmental factors dominated the relationship.

### 3.6 Path Analysis

**FIGURE 4:**
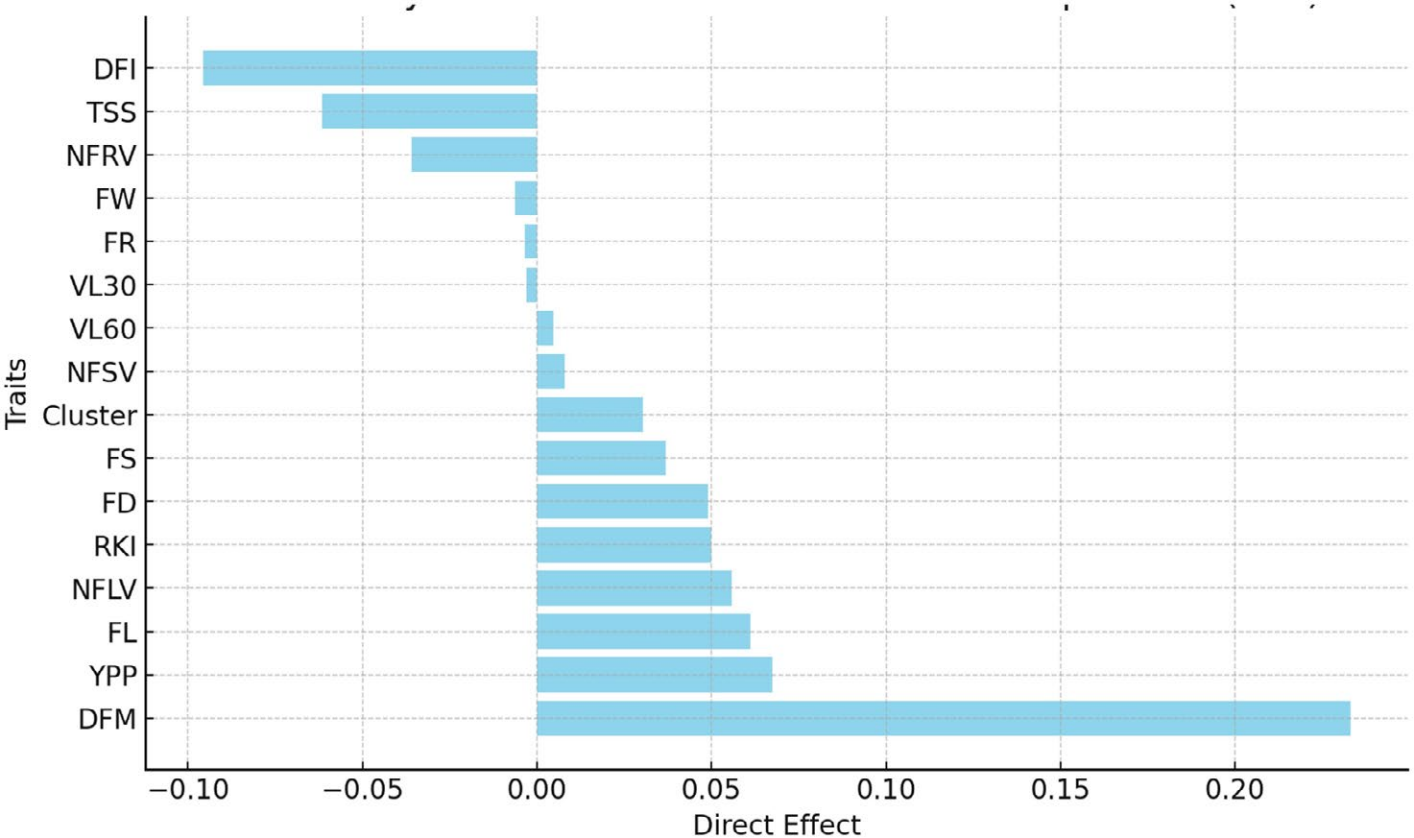
Path analysis: Direct effect of traits on yield per vine.

Path coefficient analysis can effectively resolve these correlations by determining the direct contributions of various traits to yield per vine. According to the path coefficient analysis (Figure 4), traits such as DFM, YPP, FL, NFLV, RKI, FD, FS, and Cluster showed strongly positive direct effects, while NFSV and VL 60 exhibited slightly positive direct effects on yield per plant, underscoring their roles as significant yield‐contributing factors. In contrast, traits like VL 30, FR, FW, NFRV, TSS, and DFI reported negative direct effects on yield per plant.

### 3.7 Comparison Among the Different Traits of Cucumber

The Table 5 represents a comparison of key cucumber traits between 2020 and 2021, with a focus on their mean values, ranges, coefficients of variation (CV), F‐ratios, and probabilities (*p*‐values). Significant differences were observed in traits like DFI, NFSV, and DFM, with DFI increasing significantly from 33.25 in 2020 to 35.22 in 2021 and NFSV decreasing from 26.06 to 22.91. These results suggest that environmental factors or treatment effects may have influenced these traits, leading to notable year‐to‐year variations. On the other hand, traits such as VL30, FS, and Y/M^2^ showed no significant differences, indicating consistency across the 2 years.

**TABLE 5:** Comparison of Cucumber Traits Between 2020 and 2021 Under Protected Cultivation.

Trait	2020			2021			*F*‐ratio	Probability
	Mean	Range	CV (%)	Mean 2	Range	CV (%)		
VL30	82.87^a^	43.79–129.35	29.44	75.76^a^	40.33–112.99	29.33	1.8124	0.1822
VL60	174.04^a^	118.88–268.8	19.8	162.24^a^	122.85–207.69	14.42	3.1288	0.0809
DFI	33.25^ba^	28.71–39.09	10.05	35.22^bb^	32.01–37.94	4.37	11.2619	0.0012
NFLV	34.98^a^	27.09–40.88	11.41	33.79^a^	26.79–39.84	9.44	2.1144	0.15
NFSV	26.06^ba^	17.18–33.02	16.55	22.91^bb^	15.56–29.92	17.46	11.2031	0.0013
FS	68.99^a^	48.64–91.53	15.65	67.62^a^	51.72–84.47	12.41	0.3944	0.5319
DFM	8.39^ca^	6.74–9.6	9.82	8.71^cb^	7.77–9.68	5.56	4.4065	0.0391
NFRV	18.71^a^	10.27–29.15	25.21	18.30^a^	11.42–26.12	23.39	0.1668	0.6841
FR	81.98^a^	62.98–97.87	11.92	80.51^a^	65.67–94.6	9.54	0.5418	0.464
FL	17.74^a^	15.31–23.19	10.62	17.70^a^	15.41–21.56	8.62	0.0097	0.9218
FD	4.28^aa^	3.4–5.33	11.91	3.86^ab^	3.3–4.62	8.63	18.4109	0.0001
FW	155.75^a^	109.7–232.93	17.35	153.36^a^	119.88–211.4	15.03	0.1771	0.6751
YPV	3.34^a^	1.54–4.91	24.78	3.44^a^	2.0–4.73	19.5	0.3897	0.5343
YPP	36.27a	17.41–55.61	24.46	36.67^a^	24.0–49.24	18.66	0.0491	0.8253
Y/M2	10.39^a^	4.92–15.03	23.86	10.19^a^	6.26–14.16	19.24	0.1645	0.6862
TSS	2.92^aa^	2.68–3.16	4.27	3.11^ab^	2.9–3.35	4.03	41.5321	0.0
RKI	0.44^a^	0.0–3.33	205.64	0.45^a^	0.0–2.12	164.07	0.005	0.9441
Yield (kg/m^2^)	10.52^a^	4.97–15.27	23.93	9.94^a^	6.09–14.38	19.7	1.2915	0.2593

*Note:* Columns present the mean, range, coefficient of variation (CV), F‐ratio, and probability (*p*‐value) for each trait. Mean values followed by the same second lowercase letter (e.g., aa–aa, ba–ba, or ca–ca) within a row are not significantly different between 2020 and 2021, whereas different second lowercase letters (aa–ab, ba–bb, or ca–cb) indicate a significant difference between years (*p* 〈 0.05). The first lowercase letter (a, b, or c) denotes the significance category based on the *p*‐value: a = non‐significant (*p* ≥ 0.05), b = significant (*p* 〈 0.05), and c = highly significant (*p* 〈 0.01).

The coefficients of variation (CV) highlight the variability within each year. For instance, RKI exhibited a high CV of 205.64 in 2020, indicating substantial variation, while other traits like TSS had more stable values. Traits with high F‐ratios and low *p*‐values, such as DFI and NFSV, showed strong evidence of year‐to‐year differences. Conversely, traits like RKI and Yield (kg/m^2^) had high *p*‐values, indicating no significant variation between the years. These findings suggest that while some traits respond to environmental changes, others remain stable, and understanding these patterns can help in optimizing cucumber cultivation practices and breeding strategies.

## 4 Discussion

The present study clearly demonstrated that growing media significantly influenced vegetative growth, yield, fruit quality, and root health of cucumber under protected cultivation. The superior performance of the vermiculite + cocopeat (1:1) substrate observed across both seasons is consistent with earlier reports that emphasize the role of balanced aeration and water‐holding capacity in enhancing root growth and nutrient uptake in cucurbits. The results of Al‐Far et al. (2019), who noted improved plant growth in soilless media, are consistent with this discovery. As previously mentioned by Ghehsareh and Kalbasi (2012), the improved water‐holding capacity and aeration offered by vermiculite and cocopeat may be the cause of the longer vines in T9 (Vermiculite + Cocopeat in 1:1 ratio). The complementing physical characteristics of the two substrates may be the reason for the better vegetative development seen in the Vermiculite + Cocopeat (1:1) treatment. Vermiculite enhances moisture retention, aeration, and nutritional buffering, while cocopeat offers high water‐holding capacity and cation exchange capacity, guaranteeing a steady supply of water and nutrients to the root system. When combined, these characteristics produce a favorable root‐zone environment that encourages root growth, nutrient uptake, and photosynthetic activity, leading to increased biomass accumulation and vine elongation. Similar findings have been documented in greenhouse cucumber and other vegetable crops, where vegetative growth was enhanced by balanced substrate aeration and moisture availability (Gruda 2019). Similar improvements in vine length, fruit number, and yield under cocopeat‐based media have also been reported by Abou‐Hadid et al. (1995), Medany et al. (1995), Gruda, Gallegos‐Cedillo, et al. (2025), (Gruda, Samuolienė, et al. (2025)), supporting our findings.

However, variations in magnitude among studies may be attributed to differences in substrate composition, irrigation scheduling, nutrient formulation, and microclimatic conditions within protected structures. In the present investigation, yield and its component traits exhibited relatively low heritability despite moderate to high phenotypic variability. This indicates a stronger influence of environmental factors rather than genetic control. Under protected cultivation, yield expression is highly sensitive to microclimatic fluctuations such as temperature, relative humidity, and light intensity, as well as nutrient dynamics and moisture availability within different substrates. Even minor variations in substrate water retention and nutrient release can significantly affect fruit set, retention, and final yield, thereby reducing heritability estimates for yield‐related traits (Gruda, Samuolienė, et al. 2025) and (Pooyeh et al. 2012).

The findings showed that the T9 (Vermiculite + Cocopeat in 1:1 ratio) treatment had the lowest number of days needed for flower initiation and fruit setting. Early blooming was probably caused by improved root development and nutrient availability made possible by the combination of vermiculite and cocopeat (Mazahreh et al. 2015). Similarly, Singh et al. (2017) emphasized the importance of nutrient availability in protected cucumber cultivation, corroborating the improved flower and fruit set in optimized media.

The higher fruit yield obtained in the Vermiculite + Cocopeat substrate was likely a consequence of improved root development, efficient water and nutrient uptake, and sustained physiological activity throughout the cropping period. Balanced substrate aeration reduces root‐zone hypoxia, while adequate moisture retention minimizes plant water stress, thereby enhancing photosynthesis, flower retention, fruit set, and assimilate partitioning towards developing fruits (Fesendouz et al. 2025; Singh et al. 2026). Good WHC and aeration greatly influenced the root physiology by creating the congenial micro environment for root growth and development. According to He et al. (2021), the combination of vermiculite and cocopeat probably increased the efficiency of nutrient uptake. According to Gashgari et al. (2018), soilless growth media maximize resource usage and increase fruit yield. This finding is consistent with their findings. Additionally, the fruit's length, diameter, and average weight responded favorably to the optimized media, supporting Gruda, Gallegos‐Cedillo, et al. (2025) prior findings regarding the advantages of cocopeat‐based substrates.

Soil‐alone (T1) and sand‐alone (T5) treatments showed the largest root‐knot infestation, whereas combinations of soilless media showed the least amount of infestation, which might be due to the absence of primary inoculation of nematodes. It is not appropriate to interpret the decreased RKI seen in substrate blends with larger percentages of perlite, vermiculite, and cocopeat as proof that these substrates transmit nematodes. While fresh cocopeat is usually devoid of root‐knot nematodes unless contaminated or reused, perlite and vermiculite are inert, sterile minerals that do not act as sources of nematode inoculum. The favorable physical characteristics of these substrates, such as better aeration, drainage, moisture balance, and improved root‐zone conditions, which support healthy root development and may lessen root susceptibility to nematode infection, are more likely to be responsible for the decreased RKI. On the other hand, leftover nematode inoculum is more likely to be present in substrate blends comprising soil or recycled organic materials, which raises the possibility of root colonization and disease development. These results are consistent with those of Dhillon et al. (2022), who highlighted that nematode infestations are more likely to occur in soil‐based farming. The lack of noticeable infestation in T9 indicates that soil‐borne illnesses can be successfully reduced by substituting different substrates for soil (El‐Aidy et al. 2007; Singh et al. 2019; Sahil et al. 2023; Ziaf, Ahmad, et al. 2021).

Significant correlations between important cucumber features were shown by correlation analysis conducted during both the years. It made it more likely to identify varieties and promising lines using phenotypic features (Yadav, Gangadhara, Singh, 2024; Yadav et al. 2025; Mishra et al. 2026). In 2020, the number of fruit sets per vine (NFSV) (*r* = 0.77) and yield per vine (YPV) (*r* = 0.74) showed a substantial positive connection with vine length at 30 days (VL30). Similar relationships between VL60 and fruit weight (FW) (*r* = 0.86) and number of fruits per vine (NFRV) (*r* = 0.85) were found, confirming Papadopoulos's (2001) results that fruit output is positively impacted by greater vegetative growth. On the other hand, in both years, the root‐knot infestation index (RKI) was negatively correlated with yield metrics.

Vine length, fruit set, and fruit production were the most significant features in the PCA. This is in line with Wright (1921), who emphasized how PCA helps discover important features and reduce dimensionality. Important information about the genetic and environmental factors influencing trait variability was revealed by the estimations of variance components (Yadav, Gangadhara, Apparao, et al. 2024). Strong genetic control over variables like days to flowering initiation (DFI), total soluble solids (TSS), and days to first male flowering (DFM) was demonstrated by their high heritability (> 85%). Johnson and Wichern (2007) highlighted the importance of high heritability in breeding programs, and these results are consistent with their findings.

Fruit set (FS) and fruit retention (FR) showed moderate heritability, indicating a balanced impact of environmental and genetic factors. This confirms the findings of Wright (1921). On the other hand, characteristics such as yield per plant (YPP) and yield per vine (YPV) showed low heritability (< 20%), suggesting that environmental factors dominated the relationship (Singh et al. 2018).

When 2020 and 2021 findings were compared, it was shown that environmental conditions had a substantial impact on features like fruit setting and days to bloom initiation. Singh et al. (2018) also reported on the effect of climate variations on cucumber production, and they proposed that microclimate fluctuations are important in protected farming. High heritability traits, such as fruit weight and vine length, performed consistently in both years, indicating the possibility of genetic improvement (Johnson and Wichern 2007).

From a sustainability perspective, the use of cocopeat‐ and vermiculite‐based substrates offers multiple advantages under protected cultivation, including improved water‐use efficiency, reduced soil‐borne disease incidence, and better root health, as evidenced by the lower root‐knot nematode index recorded in these treatments. These substrates also facilitate uniform fertigation and reduce nutrient losses through leaching, contributing to environmentally sustainable and resource‐efficient vegetable production systems (Abou‐Hadid et al. 1995; Pooyeh et al. 2012; Medany et al. 1995). Thus, the present findings reinforce the suitability of optimized soilless media combinations as a sustainable strategy for enhancing cucumber productivity under protected cultivation.

## 5 Conclusion

This study demonstrated that the composition of soilless growing media significantly influenced cucumber growth, fruit yield, fruit quality, and root‐knot nematode incidence under protected cultivation. The Vermiculite + Cocopeat (1:1) medium continuously outperformed the other substrate combinations in terms of vegetative growth, marketable production, fruit quality, and root‐knot nematode infestation. These results suggest that under protected cultivation, improving substrate composition can improve root health and crop output at the same time.

Practically speaking, the Vermiculite + Cocopeat (1:1) substrate can be suggested as an appropriate growing medium for protected cucumber development when increased root‐zone health and good productivity are desired. Future research should assess how soilless substrates interact with root‐knot nematode populations in various environmental settings and protected cultivation systems, as well as their long‐term stability, economic viability, and reusability.

## Funding

The authors indicate that there was no funding provided for the research presented in this article.

## Ethics Statement

No research involving human participants or animals was conducted; therefore, ethics approval was not required.

## Conflicts of Interest

The authors declare no conflicts of interest.

## Supporting information


**Data S1:** On growth, yield, fruit quality, and root‐knot nematode‐related traits of cucumber grown under protected cultivation during 2020 and 2021.

## Data Availability

The data that supports the findings of this study are available in the supporting information of this article.
