# Supplementary material for: Impact of Soilless Growing Media on Growth, Yield, Fruit Quality, and Root‐Knot Nematode Incidence of Cucumber Under Protected Cultivation
Source: Plant Environ Interact. 2026 Aug 3;7(4):e70196. doi: 10.1002/pei3.70196 (PMC13430664; doi:10.1002/pei3.70196)
Supplement: Supplementary file 1 — Data S1: On growth, yield, fruit quality, and root‐knot nematode‐related traits of cucumber grown under protected cultivation during 2020 and 2021. [file PEI3-7-e70196-s001.docx]

Supplementary dataset S1: on growth, yield, fruit quality, and root-knot nematode-related traits of cucumber grown under protected cultivation during 2020 and 2021.

| 2020 | | | | | | | | | | | | | | | | | | | | | | | | | | | | | | | | | |
| --- | --- | --- | --- | --- | --- | --- | --- | --- | --- | --- | --- | --- | --- | --- | --- | --- | --- | --- | --- | --- | --- | --- | --- | --- | --- | --- | --- | --- | --- | --- | --- | --- | --- |
| Tr | VL30 | | VL60 | | DFI | | NFV | | NFSV | | FS | | DFM | | NFV.1 | | FR | | FL | | FD | FW | YPV | YPP | | Y/M2 | | TSS | | RKI | | Yield | |
| T1 | 100.66 | | 144.24 | | 37.40 | | 35.42 | | 26.40 | | 80.69 | | 9.27 | | 16.26 | | 86.65 | | 21.18 | | 3.63 | 151.04 | 3.47 | 37.67 | | 8.62 | | 2.87 | | 3.33 | | 8.71 | |
| T2 | 58.89 | | 123.41 | | 35.62 | | 34.85 | | 19.71 | | 61.08 | | 8.99 | | 11.04 | | 78.89 | | 18.14 | | 3.40 | 124.49 | 2.18 | 24.44 | | 5.59 | | 2.99 | | 0.00 | | 5.65 | |
| T3 | 103.24 | | 179.21 | | 37.48 | | 39.48 | | 30.28 | | 82.99 | | 8.72 | | 19.98 | | 92.84 | | 19.24 | | 3.79 | 170.34 | 3.69 | 43.03 | | 9.84 | | 2.95 | | 0.00 | | 9.94 | |
| T4 | 118.99 | | 166.77 | | 38.19 | | 40.11 | | 31.62 | | 85.30 | | 8.80 | | 20.88 | | 92.90 | | 20.93 | | 4.17 | 174.77 | 4.21 | 47.42 | | 10.85 | | 3.15 | | 0.00 | | 10.96 | |
| T5 | 56.70 | | 126.16 | | 37.28 | | 36.82 | | 23.92 | | 70.29 | | 8.58 | | 14.46 | | 85.00 | | 19.46 | | 3.49 | 129.20 | 3.12 | 36.40 | | 8.33 | | 2.85 | | 2.22 | | 8.42 | |
| T6 | 96.58 | | 167.47 | | 36.80 | | 37.76 | | 28.29 | | 81.02 | | 8.88 | | 18.35 | | 91.27 | | 19.68 | | 3.56 | 152.15 | 4.05 | 45.80 | | 10.48 | | 3.03 | | 0.00 | | 10.58 | |
| T7 | 94.17 | | 175.35 | | 38.97 | | 35.78 | | 23.49 | | 71.05 | | 9.35 | | 13.70 | | 82.10 | | 19.97 | | 3.70 | 148.55 | 3.16 | 36.90 | | 8.45 | | 3.03 | | 0.00 | | 8.53 | |
| T8 | 52.19 | | 118.88 | | 37.74 | | 33.50 | | 18.30 | | 59.09 | | 9.35 | | 10.27 | | 79.00 | | 18.36 | | 3.42 | 112.80 | 1.90 | 21.49 | | 4.92 | | 3.16 | | 1.11 | | 4.97 | |
| T9 | 129.35 | | 218.45 | | 36.35 | | 39.06 | | 33.02 | | 91.53 | | 8.22 | | 22.96 | | 97.87 | | 23.19 | | 4.30 | 197.41 | 4.91 | 55.61 | | 12.72 | | 2.95 | | 0.00 | | 12.85 | |
| T10 | 114.41 | | 191.10 | | 37.96 | | 38.73 | | 28.45 | | 79.51 | | 8.99 | | 19.20 | | 95.03 | | 19.54 | | 3.66 | 140.64 | 3.86 | 42.97 | | 9.83 | | 3.05 | | 0.00 | | 9.93 | |
| T11 | 61.29 | | 148.16 | | 39.01 | | 36.36 | | 25.61 | | 76.23 | | 8.94 | | 15.79 | | 86.71 | | 19.24 | | 3.52 | 134.04 | 3.66 | 42.67 | | 9.77 | | 3.16 | | 0.00 | | 9.86 | |
| T12 | 77.90 | | 157.68 | | 39.09 | | 37.25 | | 29.53 | | 85.80 | | 9.60 | | 19.41 | | 92.52 | | 18.47 | | 3.90 | 140.47 | 4.22 | 46.77 | | 10.71 | | 3.10 | | 0.00 | | 10.81 | |
| T13 | 79.97 | | 165.39 | | 37.87 | | 37.29 | | 30.54 | | 88.66 | | 8.77 | | 20.39 | | 93.98 | | 19.75 | | 3.86 | 150.95 | 4.28 | 48.01 | | 10.99 | | 3.05 | | 0.00 | | 11.10 | |
| T1 | 99.17 | | 148.44 | | 31.47 | | 28.64 | | 25.67 | | 67.16 | | 9.18 | | 16.38 | | 69.18 | | 17.94 | | 4.40 | 146.89 | 3.39 | 30.52 | | 10.18 | | 2.75 | | 2.63 | | 10.15 | |
| T2 | 58.02 | | 127.01 | | 29.97 | | 28.18 | | 19.16 | | 50.84 | | 8.90 | | 11.12 | | 62.98 | | 15.37 | | 4.13 | 121.07 | 2.13 | 19.80 | | 6.60 | | 2.87 | | 0.00 | | 6.59 | |
| T3 | 101.72 | | 184.44 | | 31.53 | | 31.93 | | 29.44 | | 69.08 | | 8.64 | | 20.13 | | 74.11 | | 16.30 | | 4.59 | 165.66 | 3.60 | 34.86 | | 11.62 | | 2.82 | | 0.00 | | 11.59 | |
| T4 | 117.24 | | 171.63 | | 32.13 | | 32.44 | | 30.74 | | 71.00 | | 8.71 | | 21.04 | | 74.17 | | 17.73 | | 5.06 | 169.96 | 4.12 | 38.42 | | 12.82 | | 3.02 | | 0.00 | | 12.78 | |
| T5 | 55.87 | | 129.84 | | 31.37 | | 29.78 | | 23.25 | | 58.50 | | 8.50 | | 14.57 | | 67.86 | | 16.48 | | 4.23 | 125.65 | 3.05 | 29.50 | | 9.84 | | 2.73 | | 1.75 | | 9.81 | |
| T6 | 95.15 | | 172.35 | | 30.97 | | 30.54 | | 27.50 | | 67.43 | | 8.79 | | 18.48 | | 72.86 | | 16.67 | | 4.32 | 147.97 | 3.96 | 37.11 | | 12.38 | | 2.91 | | 0.00 | | 12.34 | |
| T7 | 92.78 | | 180.47 | | 32.79 | | 28.93 | | 22.84 | | 59.14 | | 9.26 | | 13.80 | | 65.55 | | 16.92 | | 4.49 | 144.47 | 3.09 | 29.90 | | 9.97 | | 2.91 | | 0.00 | | 9.95 | |
| T8 | 51.42 | | 122.34 | | 31.75 | | 27.09 | | 17.79 | | 49.18 | | 9.26 | | 10.34 | | 63.07 | | 15.55 | | 4.15 | 109.70 | 1.86 | 17.41 | | 5.81 | | 3.03 | | 0.88 | | 5.79 | |
| T9 | 127.45 | | 224.81 | | 30.59 | | 31.59 | | 32.10 | | 76.19 | | 8.14 | | 23.13 | | 78.13 | | 19.65 | | 5.22 | 191.98 | 4.80 | 45.06 | | 15.03 | | 2.82 | | 0.00 | | 14.98 | |
| T10 | 112.72 | | 196.67 | | 31.94 | | 31.32 | | 27.66 | | 66.18 | | 8.90 | | 19.35 | | 75.86 | | 16.55 | | 4.45 | 136.77 | 3.77 | 34.82 | | 11.61 | | 2.92 | | 0.00 | | 11.58 | |
| T11 | 60.39 | | 152.48 | | 32.82 | | 29.41 | | 24.90 | | 63.45 | | 8.86 | | 15.90 | | 69.22 | | 16.30 | | 4.28 | 130.36 | 3.58 | 34.58 | | 11.54 | | 3.03 | | 0.00 | | 11.50 | |
| T12 | 76.75 | | 162.28 | | 32.89 | | 30.13 | | 28.70 | | 71.42 | | 9.51 | | 19.55 | | 73.86 | | 15.65 | | 4.73 | 136.61 | 4.13 | 37.90 | | 12.64 | | 2.97 | | 0.00 | | 12.61 | |
| T13 | 78.79 | | 170.22 | | 31.87 | | 30.15 | | 29.69 | | 73.79 | | 8.68 | | 20.55 | | 75.03 | | 16.74 | | 4.68 | 146.80 | 4.18 | 38.90 | | 12.98 | | 2.92 | | 0.00 | | 12.94 | |
| T1 | 84.45 | | 177.49 | | 30.14 | | 36.10 | | 24.78 | | 66.43 | | 7.60 | | 20.65 | | 84.08 | | 17.87 | | 4.49 | 178.23 | 2.81 | 32.51 | | 10.04 | | 2.70 | | 2.65 | | 10.35 | |
| T2 | 49.41 | | 151.86 | | 28.71 | | 35.52 | | 18.50 | | 50.28 | | 7.37 | | 14.01 | | 76.55 | | 15.31 | | 4.21 | 146.89 | 1.76 | 21.09 | | 6.52 | | 2.81 | | 0.00 | | 6.71 | |
| T3 | 86.62 | | 220.53 | | 30.21 | | 40.24 | | 28.42 | | 68.32 | | 7.15 | | 25.37 | | 90.08 | | 16.23 | | 4.69 | 201.00 | 2.98 | 37.14 | | 11.47 | | 2.77 | | 0.00 | | 11.81 | |
| T4 | 99.84 | | 205.21 | | 30.78 | | 40.88 | | 29.68 | | 70.22 | | 7.21 | | 26.51 | | 90.15 | | 17.66 | | 5.17 | 206.22 | 3.41 | 40.93 | | 12.64 | | 2.96 | | 0.00 | | 13.02 | |
| T5 | 47.57 | | 155.25 | | 30.05 | | 37.52 | | 22.45 | | 57.86 | | 7.03 | | 18.36 | | 82.48 | | 16.42 | | 4.32 | 152.45 | 2.52 | 31.42 | | 9.71 | | 2.68 | | 1.77 | | 10.00 | |
| T6 | 81.03 | | 206.08 | | 29.66 | | 38.49 | | 26.55 | | 66.70 | | 7.28 | | 23.30 | | 88.56 | | 16.60 | | 4.41 | 179.53 | 3.28 | 39.53 | | 12.21 | | 2.85 | | 0.00 | | 12.58 | |
| T7 | 79.01 | | 215.78 | | 31.41 | | 36.46 | | 22.05 | | 58.49 | | 7.66 | | 17.40 | | 79.67 | | 16.85 | | 4.58 | 175.28 | 2.56 | 31.85 | | 9.84 | | 2.85 | | 0.00 | | 10.13 | |
| T8 | 43.79 | | 146.28 | | 30.42 | | 34.15 | | 17.18 | | 48.64 | | 7.66 | | 13.04 | | 76.65 | | 15.49 | | 4.23 | 133.10 | 1.54 | 18.55 | | 5.73 | | 2.97 | | 0.88 | | 5.90 | |
| T9 | 108.53 | | 268.80 | | 29.30 | | 39.81 | | 31.00 | | 75.35 | | 6.74 | | 29.15 | | 94.97 | | 19.56 | | 5.33 | 232.93 | 3.98 | 47.99 | | 14.82 | | 2.77 | | 0.00 | | 15.27 | |
| T10 | 95.99 | | 235.15 | | 30.60 | | 39.47 | | 26.71 | | 65.46 | | 7.37 | | 24.38 | | 92.21 | | 16.48 | | 4.54 | 165.95 | 3.13 | 37.09 | | 11.46 | | 2.86 | | 0.00 | | 11.80 | |
| T11 | 51.42 | | 182.31 | | 31.44 | | 37.06 | | 24.04 | | 62.76 | | 7.33 | | 20.05 | | 84.14 | | 16.23 | | 4.36 | 158.16 | 2.97 | 36.83 | | 11.38 | | 2.97 | | 0.00 | | 11.72 | |
| T12 | 65.36 | | 194.03 | | 31.51 | | 37.97 | | 27.71 | | 70.64 | | 7.87 | | 24.64 | | 89.77 | | 15.58 | | 4.83 | 165.75 | 3.42 | 40.37 | | 12.47 | | 2.91 | | 0.00 | | 12.85 | |
| T13 | 67.09 | | 203.52 | | 30.52 | | 38.00 | | 28.67 | | 72.99 | | 7.19 | | 25.90 | | 91.19 | | 16.67 | | 4.78 | 178.12 | 3.46 | 41.44 | | 12.80 | | 2.86 | | 0.00 | | 13.19 | |
| 2021 | | | | | | | | | | | | | | | | | | | | | | | | | | | | | | | | | |
| Tr | VL30 | VL60 | | DFI | | NFV | | NFSV | | FS% | | DFM | | NFV.1 | | FR% | | FL | | FD | | FW | YPV | | YPP | | Y/M2 | | TSS | | RKI | | Yield |
| T1 | 87.73 | 145.32 | | 36.04 | | 29.42 | | 21.99 | | 62.81 | | 9.04 | | 15.26 | | 72.68 | | 19.14 | | 3.67 | | 146.23 | 3.11 | | 31.63 | | 8.97 | | 3.06 | | 1.86 | | 9.24 |
| T2 | 56.38 | 127.74 | | 36.29 | | 29.59 | | 18.23 | | 51.93 | | 9.26 | | 11.42 | | 65.67 | | 17.09 | | 3.44 | | 141.76 | 2.21 | | 26.13 | | 7.42 | | 3.08 | | 0.00 | | 7.64 |
| T3 | 99.34 | 180.08 | | 35.89 | | 34.57 | | 26.59 | | 64.67 | | 8.17 | | 19.79 | | 77.98 | | 19.62 | | 3.99 | | 190.96 | 3.46 | | 39.21 | | 11.13 | | 3.06 | | 0.00 | | 11.46 |
| T4 | 112.99 | 186.36 | | 34.98 | | 35.32 | | 29.16 | | 69.40 | | 8.82 | | 22.15 | | 79.63 | | 19.27 | | 3.97 | | 192.91 | 3.74 | | 41.92 | | 11.89 | | 3.09 | | 0.00 | | 12.25 |
| T5 | 46.81 | 132.22 | | 36.98 | | 30.83 | | 20.11 | | 54.90 | | 9.44 | | 13.21 | | 68.75 | | 16.46 | | 3.30 | | 137.62 | 2.84 | | 31.52 | | 8.94 | | 3.07 | | 1.86 | | 9.21 |
| T6 | 85.22 | 162.31 | | 36.10 | | 31.72 | | 24.68 | | 65.46 | | 8.87 | | 17.65 | | 74.89 | | 17.00 | | 3.64 | | 173.31 | 3.47 | | 41.32 | | 11.73 | | 3.06 | | 0.00 | | 12.08 |
| T7 | 81.92 | 166.66 | | 36.55 | | 28.53 | | 21.34 | | 62.88 | | 8.93 | | 13.65 | | 67.04 | | 17.22 | | 3.73 | | 154.76 | 2.96 | | 33.64 | | 9.54 | | 3.15 | | 0.00 | | 9.83 |
| T8 | 42.90 | 122.85 | | 37.94 | | 26.79 | | 18.09 | | 56.74 | | 9.33 | | 11.49 | | 66.60 | | 16.25 | | 3.41 | | 135.06 | 2.00 | | 24.16 | | 6.86 | | 3.21 | | 0.93 | | 7.06 |
| T9 | 111.27 | 197.16 | | 36.53 | | 34.83 | | 29.92 | | 72.24 | | 8.28 | | 23.70 | | 83.06 | | 21.56 | | 4.08 | | 211.40 | 4.27 | | 49.24 | | 13.96 | | 3.03 | | 0.00 | | 14.38 |
| T10 | 97.28 | 177.29 | | 36.62 | | 32.69 | | 26.02 | | 66.87 | | 8.76 | | 19.16 | | 77.18 | | 17.76 | | 3.58 | | 164.34 | 3.58 | | 40.26 | | 11.43 | | 3.16 | | 0.93 | | 11.77 |
| T11 | 54.43 | 144.45 | | 36.35 | | 30.34 | | 23.00 | | 63.73 | | 9.68 | | 15.51 | | 70.68 | | 17.88 | | 3.42 | | 151.34 | 3.34 | | 37.00 | | 10.49 | | 3.13 | | 0.00 | | 10.81 |
| T12 | 72.11 | 157.11 | | 36.20 | | 31.62 | | 26.27 | | 69.81 | | 9.35 | | 19.14 | | 76.24 | | 17.23 | | 3.73 | | 152.86 | 3.73 | | 40.33 | | 11.44 | | 3.14 | | 0.00 | | 11.78 |
| T13 | 70.53 | 159.23 | | 36.25 | | 32.78 | | 27.96 | | 71.71 | | 8.33 | | 20.90 | | 78.32 | | 19.62 | | 3.88 | | 176.88 | 3.75 | | 42.02 | | 11.92 | | 3.15 | | 0.00 | | 12.28 |
| T1 | 82.48 | 153.09 | | 35.71 | | 32.18 | | 18.92 | | 62.55 | | 8.79 | | 16.82 | | 82.58 | | 18.68 | | 4.15 | | 134.68 | 3.28 | | 31.52 | | 9.10 | | 3.20 | | 2.12 | | 7.97 |
| T2 | 53.01 | 134.57 | | 35.97 | | 32.37 | | 15.68 | | 51.72 | | 9.00 | | 12.59 | | 74.61 | | 16.68 | | 3.89 | | 130.57 | 2.34 | | 26.04 | | 7.52 | | 3.22 | | 0.00 | | 6.59 |
| T3 | 93.39 | 189.70 | | 35.57 | | 37.81 | | 22.87 | | 64.40 | | 7.94 | | 21.81 | | 88.61 | | 19.15 | | 4.51 | | 175.89 | 3.65 | | 39.07 | | 11.28 | | 3.20 | | 0.00 | | 9.88 |
| T4 | 106.22 | 196.32 | | 34.67 | | 38.63 | | 25.08 | | 69.11 | | 8.58 | | 24.41 | | 90.48 | | 18.80 | | 4.49 | | 177.68 | 3.95 | | 41.77 | | 12.06 | | 3.23 | | 0.00 | | 10.56 |
| T5 | 44.00 | 139.29 | | 36.65 | | 33.73 | | 17.30 | | 54.68 | | 9.18 | | 14.55 | | 78.11 | | 16.07 | | 3.74 | | 126.75 | 2.99 | | 31.40 | | 9.07 | | 3.21 | | 2.12 | | 7.94 |
| T6 | 80.12 | 170.98 | | 35.78 | | 34.69 | | 21.23 | | 65.19 | | 8.62 | | 19.46 | | 85.09 | | 16.59 | | 4.12 | | 159.63 | 3.66 | | 41.18 | | 11.89 | | 3.20 | | 0.00 | | 10.42 |
| T7 | 77.01 | 175.57 | | 36.22 | | 31.20 | | 18.35 | | 62.63 | | 8.68 | | 15.04 | | 76.17 | | 16.80 | | 4.22 | | 142.55 | 3.13 | | 33.52 | | 9.68 | | 3.29 | | 0.00 | | 8.47 |
| T8 | 40.33 | 129.42 | | 37.60 | | 29.31 | | 15.56 | | 56.51 | | 9.07 | | 12.66 | | 75.67 | | 15.86 | | 3.86 | | 124.39 | 2.11 | | 24.08 | | 6.96 | | 3.35 | | 1.06 | | 6.09 |
| T9 | 104.61 | 207.69 | | 36.20 | | 38.09 | | 25.73 | | 71.94 | | 8.05 | | 26.12 | | 94.37 | | 21.05 | | 4.62 | | 194.72 | 4.51 | | 49.07 | | 14.16 | | 3.17 | | 0.00 | | 12.40 |
| T10 | 91.45 | 186.76 | | 36.29 | | 35.76 | | 22.38 | | 66.60 | | 8.51 | | 21.12 | | 87.69 | | 17.33 | | 4.05 | | 151.37 | 3.78 | | 40.12 | | 11.59 | | 3.30 | | 1.06 | | 10.15 |
| T11 | 51.17 | 152.16 | | 36.02 | | 33.19 | | 19.78 | | 63.47 | | 9.41 | | 17.10 | | 80.31 | | 17.45 | | 3.87 | | 139.40 | 3.53 | | 36.87 | | 10.64 | | 3.27 | | 0.00 | | 9.32 |
| T12 | 67.79 | 165.50 | | 35.87 | | 34.59 | | 22.59 | | 69.53 | | 9.09 | | 21.10 | | 86.63 | | 16.82 | | 4.22 | | 140.79 | 3.94 | | 40.19 | | 11.60 | | 3.28 | | 0.00 | | 10.16 |
| T13 | 66.31 | 167.73 | | 35.92 | | 35.85 | | 24.05 | | 71.42 | | 8.10 | | 23.03 | | 88.99 | | 19.15 | | 4.39 | | 162.92 | 3.96 | | 41.87 | | 12.09 | | 3.29 | | 0.00 | | 10.59 |
| T1 | 84.18 | 148.22 | | 32.97 | | 33.19 | | 21.80 | | 73.45 | | 8.59 | | 16.75 | | 82.78 | | 18.15 | | 3.74 | | 129.80 | 3.44 | | 31.42 | | 8.19 | | 2.92 | | 1.93 | | 8.41 |
| T2 | 54.10 | 130.29 | | 33.21 | | 33.38 | | 18.06 | | 60.73 | | 8.80 | | 12.53 | | 74.79 | | 16.21 | | 3.50 | | 125.84 | 2.45 | | 25.95 | | 6.77 | | 2.94 | | 0.00 | | 6.96 |
| T3 | 95.32 | 183.68 | | 32.84 | | 38.99 | | 26.35 | | 75.62 | | 7.77 | | 21.71 | | 88.82 | | 18.61 | | 4.06 | | 169.51 | 3.83 | | 38.94 | | 10.15 | | 2.92 | | 0.00 | | 10.43 |
| T4 | 108.42 | 190.08 | | 32.01 | | 39.84 | | 28.90 | | 81.15 | | 8.38 | | 24.31 | | 90.70 | | 18.27 | | 4.05 | | 171.24 | 4.15 | | 41.63 | | 10.85 | | 2.95 | | 0.00 | | 11.15 |
| T5 | 44.91 | 134.86 | | 33.83 | | 34.78 | | 19.93 | | 64.20 | | 8.97 | | 14.49 | | 78.30 | | 15.62 | | 3.36 | | 122.16 | 3.14 | | 31.30 | | 8.16 | | 2.93 | | 1.93 | | 8.38 |
| T6 | 81.77 | 165.55 | | 33.03 | | 35.78 | | 24.46 | | 76.54 | | 8.43 | | 19.37 | | 85.30 | | 16.13 | | 3.71 | | 153.84 | 3.84 | | 41.04 | | 10.70 | | 2.92 | | 0.00 | | 11.00 |
| T7 | 78.61 | 169.99 | | 33.44 | | 32.18 | | 21.15 | | 73.53 | | 8.49 | | 14.97 | | 76.35 | | 16.33 | | 3.80 | | 137.38 | 3.28 | | 33.41 | | 8.71 | | 3.01 | | 0.00 | | 8.95 |
| T8 | 41.16 | 125.30 | | 34.71 | | 30.23 | | 17.93 | | 66.35 | | 8.87 | | 12.60 | | 75.86 | | 15.41 | | 3.48 | | 119.88 | 2.21 | | 24.00 | | 6.26 | | 3.06 | | 0.97 | | 6.43 |
| T9 | 106.77 | 201.10 | | 33.42 | | 39.28 | | 29.65 | | 84.47 | | 7.87 | | 26.01 | | 94.60 | | 20.45 | | 4.16 | | 187.66 | 4.73 | | 48.90 | | 12.75 | | 2.90 | | 0.00 | | 13.10 |
| T10 | 93.34 | 180.83 | | 33.51 | | 36.88 | | 25.78 | | 78.19 | | 8.32 | | 21.02 | | 87.91 | | 16.84 | | 3.64 | | 145.88 | 3.96 | | 39.99 | | 10.43 | | 3.02 | | 0.97 | | 10.72 |
| T11 | 52.23 | 147.33 | | 33.25 | | 34.23 | | 22.79 | | 74.52 | | 9.20 | | 17.02 | | 80.51 | | 16.96 | | 3.49 | | 134.34 | 3.70 | | 36.75 | | 9.58 | | 2.99 | | 0.00 | | 9.84 |
| T12 | 69.19 | 160.25 | | 33.12 | | 35.67 | | 26.04 | | 81.63 | | 8.89 | | 21.00 | | 86.84 | | 16.34 | | 3.80 | | 135.69 | 4.14 | | 40.05 | | 10.44 | | 3.00 | | 0.00 | | 10.73 |
| T13 | 67.67 | 162.40 | | 33.17 | | 36.97 | | 27.71 | | 83.85 | | 7.92 | | 22.93 | | 89.20 | | 18.61 | | 3.95 | | 157.01 | 4.16 | | 41.74 | | 10.88 | | 3.01 | | 0.00 | | 11.18 |
